# Associations between upper limb flexibility and all-cause mortality in the oldest-old

**DOI:** 10.7189/jogh.15.04224

**Published:** 2025-10-03

**Authors:** Yushan Zhang, Chi Zhang, Jia Hu, Yuting Kang, Jie Zhang, Jianliang Zhao, Hong Shi, Ji Shen

**Affiliations:** 1 Department of Geriatrics, Beijing Hospital, National Centre of Gerontology, Institute of Geriatric Medicine, Chinese Academy of Medical Sciences, Beijing, China; 2 The Key Laboratory of Geriatrics, Beijing Institute of Geriatrics, Institute of Geriatric Medicine, Chinese Academy of Medical Sciences, Beijing Hospital, National Centre of Gerontology of National Health Commission, Beijing, China; 3 Centre for Health Policy and Management, Institute of Medical Information & Library, Chinese Academy of Medical Sciences & Peking Union Medical College, Beijing, China; 4 Department of Science Research, Beijing Hospital, National Centre of Gerontology, Institute of Geriatric Medicine, Chinese Academy of Medical Sciences, Beijing, China; 5 School of Medical Humanities, Peking University Health Science Centre,Beijing, China

## Abstract

**Background:**

Upper limb flexibility (ULF) is a simple and feasible measure of physical function in long-lived population. This study aimed to explore the association of ULF with all-cause mortality in community-dwelling oldest-old in China.

**Methods:**

A total of 21 861 older adults aged ≥80 years were enrolled from five waves of the Chinese Longitudinal Healthy Longevity Survey. The assessment of ULF at baseline involved three objective physical tasks (touching the root of the neck, touching the lower back, and raising arms vertically). All participants were followed up until 2018, during which survival information was collected. Cox proportional hazards regression model was used to analyse the association between ULF and all-cause mortality. Demographic characteristics, health behaviours, and disease history were included as covariates.

**Results:**

After 72 586.42 person years of follow-up, 18 570 people died. After adjusting for all confounding factors, both left and right ULF impairments were correlated with an increased risk of all-cause mortality, with adjusted hazard ratio (HR) of 1.06 (95% confidence interval (CI) = 1.01, 1.12) and 1.12 (95% CI = 1.05, 1.20). In the collaborative analyses, individuals exhibiting ULF impairment across both the left and right sides had the highest mortality risk with adjusted HR of 1.29 (95% CI = 1.19, 1.38). We found significant additive interaction between left and right ULF impairment on all-cause mortality (relative excess risk due to interaction = 0.10; 95% CI = 0.02, 0.18). The principal findings maintained stable across sensitivity analyses.

**Conclusion:**

Impaired ULF is associated with a higher risk of all-cause mortality among the oldest-old in China, especially when the impairment occurs on the right side. For the oldest-old, ULF may serve as a simple and effective predictor of premature death.

According to the seventh national census of China in 2020, the number of individuals aged 80 and above was 35.8 million. This figure is projected to rise to 70.84 million by 2035 and to 135 million by 2050, positioning China as the nation with the largest older population globally [1,2]. With advancing age, the oldest-old individuals frequently experience a decline in bodily functions due to chronic diseases and other factors, leading to reduced physical strength [1], reaction time [3], coordination [4], and balance [5]. Physical function decline is likely to increase the risk of adverse outcomes such as hospitalisation [6], falls [7], disability [8], and mortality [9] in older adults [10]. Decreased motor function is one of the important predictors of mortality in the older adults [11]. For example, hand grip strength, as a simple and easy method to assess strength, has been shown to be significantly associated with mortality in community-dwelling older people [12,13]. In addition, motor function indicators such as walking speed [11], balance ability [14] and physical activity level [15] are also closely related to the risk of death. Consequently, assessing and intervening in the physical functioning of the oldest-old is crucial for delaying disability and enhancing quality of life, while also contributing to the reduction of premature mortality.

Currently, assessments of physical function among the oldest-old primarily focus on muscle strength, physical performance, and balance. Previous studies have utilised grip strength [16,17], walking speed [18], the Short Physical Performance Battery [19–21], and the one-leg standing test [22] to evaluate physical function. In large-scale population studies, the oldest-old often face mobility challenges, frailty, and chronic diseases, and may be unable to self-care. Assessments like Short Physical Performance Battery, grip strength, and walking speed require medical supervision and specialised equipment, which may exceed the physical capacity and tolerance of these individuals. Thus, there is a need to identify a simple, accessible, and cost-effective measure to assess limb function among the oldest-old. Among the various elements that constitute health, flexibility stands out as particularly crucial for individuals in the oldest-old age group, as it helps mitigate the effects of disability and distress [23]. A lack of flexibility reduces quality of life and increases the risk of falling in the oldest-old. The grip strength test primarily assesses isolated muscle group strength, whereas the mobility test emphasises lower limb function; neither metric adequately captures the comprehensive motor control of the upper limbs. Upper limb flexibility (ULF) offers distinct advantages as a marker of fine motor control, neurophysiological integrity, and functional performance in activities of daily living [24]. From a biomechanical perspective, flexibility significantly influences the range of motion of joints, which is critical for completing various functional movements. From a neurological perspective, the control of the upper limbs requires sophisticated neuromuscular coordination [25]. The cerebral cortex exerts significant control over upper limb fine motor coordination. Thus, ULF may serve as an indicator of nervous system functional integrity.

Studies have demonstrated that frail older individuals frequently exhibit motor dysfunctions, including reduced movement speed, grip strength decline, and impaired balance [26]. For instance, diminished ULF can result in decreased activity levels and heightened fatigue, potentially exacerbating frailty progression. Sarcopenia can affect the quality of life of the older people and increase the risk of falls, fractures, metabolic disorders and premature death [27]. In addition, ULF may also be affected by factors such as muscle mass and neuromuscular control. Although there are relatively few literatures directly studying the relationship between ULF and mortality, it can be inferred by integrating existing research results. First, as an important component of motor function, ULF is closely related to frailty and sarcopenia. Second, frailty and sarcopenia are important predictors of mortality in the older adults. Therefore, it can be inferred that ULF may also indirectly affect the risk of death in the older adults by affecting the occurrence and development of frailty and sarcopenia. However, the effect of limb flexibility on health outcomes has not been well studied. This study utilised data from the Chinese Longitudinal Healthy Longevity Survey (CLHLS) to investigate the association between ULF impairment and the risk of all-cause mortality among the oldest-old.

## METHODS

### Study population

**Figure 1:**
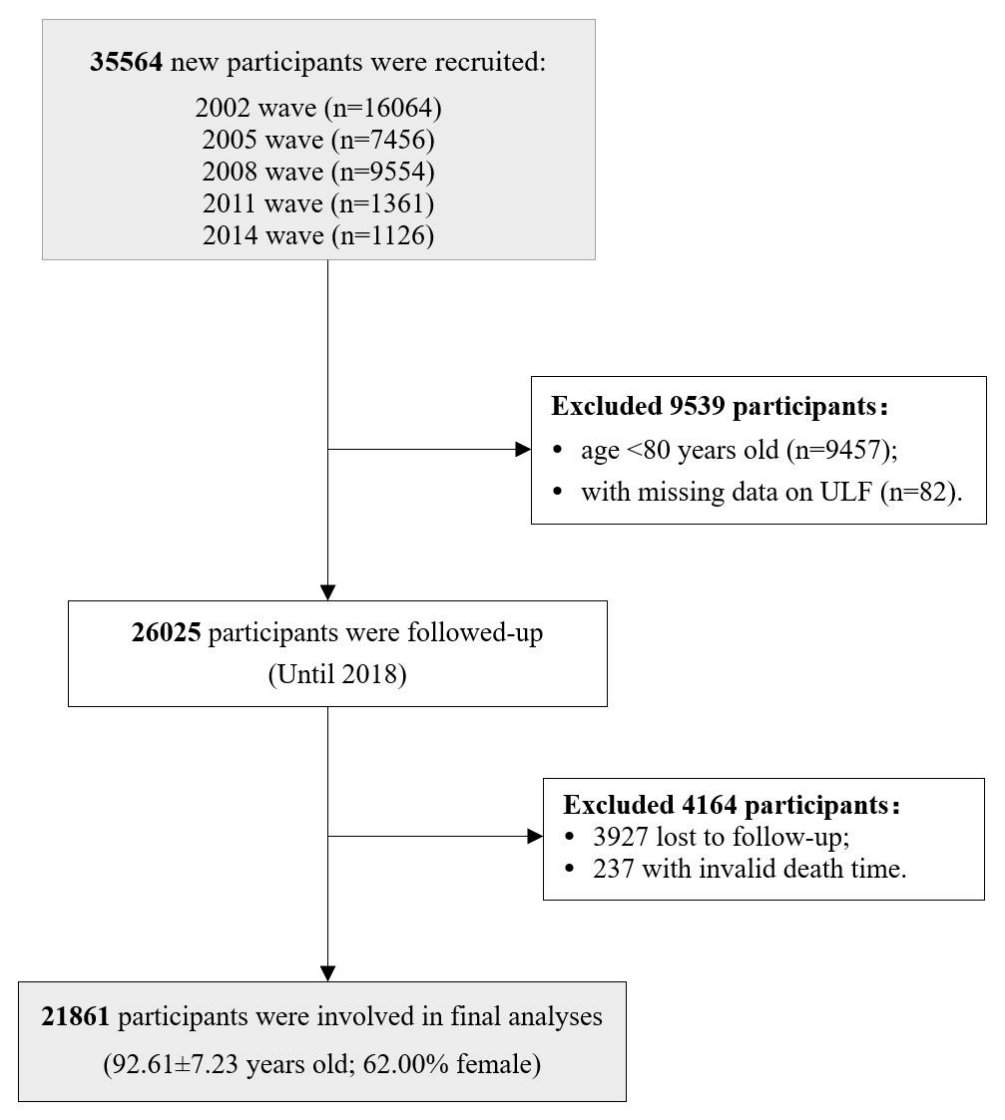
Flowchart of participant recruitment and follow-up interviews.

Study data were collected from the CLHLS, a cohort study focusing on healthy longevity led by Peking University and the Chinese Centre for Disease Control and Prevention. The data set comprised 35 564 participants selected from five waves (2002, 2005, 2008, 2011, 2014), ensuring no duplication in the research sample. The follow-up period extended through to 2018. Participants aged <80 years (n = 9457) and those with missing data on ULF (n = 82) were excluded. Additionally, 3927 participants lost to follow-up and 237 participants with invalid death time were also excluded. As a result, the final analysis encompassed a total of 21 861 individuals (**Figure 1**). We compared the basic characteristics of participants who were finally included and those who were lost to follow-up. Those who were lost to follow-up were older than included participants, and there was no statistical difference in gender between the two groups (Table S1 in the **Online Supplementary Document**).

### Upper limb flexibility

During each survey round, participants were instructed to sequentially perform three specified activity tasks using their left and right upper limbs: touching the root of the neck, touching the lower back, and raising their arms vertically. Successful performance on all three tasks indicated that a participant's ULF was normal. otherwise, it was defined as impaired. The ULF measurement had good internal consistency with a Cronbach’s α coefficient of 0.858, and the correlation coefficients between the three ULF tasks were greater than 0.65 (*P* < 0.001). The three ULF tasks utilised in this study mainly referred to the Disabilities of the Arm, Shoulder and Hand (DASH) scale and the dimension of upper limb activities from the disabilities of the arm, shoulder, and hand [28], which have been applied to the evaluation of upper limbs function in previous studies [29,30]. Consequently, participants were categorised into four groups based on the presence of ULF impairment: the normal group, left-side impairment alone, right-side impairment alone, and bilateral impairment.

### Survival information

Survival data for all participants were collected, and the time of death was documented through either the local civil affairs system or inquiries with close relatives at each follow-up interval. For those who passed away, survival time was calculated as the period from the date of inclusion to the date of death. For those still alive or censored, survival time was defined as the period from the last effective follow-up to the date of inclusion. Participants lost to follow-up during the initial tracking phase were excluded from the study due to the inability to ascertain their precise survival times.

### Covariates

Based on previous researches, the following covariates were included in the multiple Cox models:

1. Sociodemographic characteristics: age (continuous variable); gender (male or female); ethnicity (Han or other); residence (rural or urban); living status (living alone or not); and marital status (married and living with a spouse or not). Given the advanced age of participants, educational level was categorised based on whether the duration of education exceeded one year.

2. Health status and behaviours: body mass index (BMI); current smoking status (yes or no); current alcohol consumption (yes or no); and regular exercise (yes or no). Activities of daily living (ADL) were evaluated using the 6-item Katz scale and a total score <6 was classified as ADL impaired [31]. Cognitive function was assessed using the Mini-Mental State Examination (MMSE) and a total MMSE score <24 was classified as cognitive impairment [32,33].

3. History of chronic diseases: hypertension, diabetes, heart disease, cerebrovascular disease, and respiratory disease were collected through standardised self-reported questionnaires.

### Statistical analysis

Analysis of Variance (ANOVA) or the Chi-Square test was used to compare baseline characteristics. Survival probabilities across various limb flexibility groups were assessed using Kaplan-Meier survival curves and the log-rank test. Three adjusted Cox proportional hazards models were applied to examine the association between ULF and the risk of all-cause mortality:

1. Model 1 adjusted for age and sex

2. Model 2 further adjusted for residence, living arrangement, ethnicity, marital status, education, smoking, alcohol consumption, regular exercise, and BMI

3. Model 3 additionally adjusted for ADL impairment, cognitive function, heart disease, cerebrovascular disease, respiratory disease, hypertension, and diabetes.

Hazard ratios (HR) and 95% confidence intervals (CI) were calculated for each model. The Schoenfeld residual method was used to verify the proportional hazards assumption. Given that most daily activities of older individuals require coordination of both upper limbs, joint analyses were conducted to evaluate potential synergistic effects of left and right ULF impairments on mortality. Using Li et al.'s methodology for biological interaction within proportional hazards models, key metrics such as Relative Excess Risk due to Interaction (RERI), Attributable Proportion due to Interaction (AP), and Synergy Index (SI) were calculated to assess additive interactions, with significant positive interaction indicated when RERI was greater than zero and excluded zero within its 95% CI.

The following sensitivity analyses were conducted to test the stability of the primary results:

1. Considering the potential reverse causality due to early decedents, we excluded 2324 descents in the first year

2. Since severe pathological conditions may confound the association, we sequentially excluded participants who had heart disease (n = 1559) or cerebrovascular disease (n = 1064)

3. Considering that cognitive impairment and ADL decline may affect limb flexibility, we also excluded 2753 participants with severe cognitive impairment (MMSE score <14) and 4601 participants with severe physical function impairment (Katz ADL score <5) to test the stability of main results.

All statistical analyses were performed using *R* software version 4.2.0 (*R* Foundation of Statistical Computing, Vienna, Austria), with a two-tailed *P-*value <0.05 considered statistically significant.

## RESULTS

### Demographic characteristics

The mean age of the 21 861 participants was 92.61 ± 7.23 years, and 13 553 (62.00%) were female. A total of 3391 (15.51%) participants had left ULF impairment, 2638 (12.07%) had right ULF impairment, and 2196 (10.05%) had bilateral ULF impairment. Significant statistical differences were observed in the primary demographic characteristics and health status across the different ULF status groups (**Table 1**).

**Table 1:** Demographic characteristics of 21 861 oldest-old individuals across upper limb flexibility status*

Characteristics	Overall (n = 21 861)	Upper limb flexibility				t/Z/ꭓ^2^	*P*-value†
		**Normal (n = 18 030)**	**Left impaired alone (n = 1193)**	**Right impaired alone (n = 440)**	**Left + right impaired (n = 2198)**		
Age in years, x̄ (SD)	92.61 ± 7.23	91.51 ± 7.18	93.67 ± 7.11	93.59 ± 7.18	96.59 ± 6.67	219.85	<0.001
Female	13 553 (62.00)	10 817 (59.99)	786 (65.88)	311 (70.68)	1639 (74.57)	209.01	<0.001
Han ethnic	20 442 (93.51)	16 815 (93.26)	1123 (94.13)	427 (97.05)	2077 (94.49)	17.53	0.001
Rural or town	17 704 (80.98)	14 664 (81.33)	934 (78.29)	340 (77.27)	1766 (80.35)	11.19	0.012
Live alone	3305 (15.12)	2839 (15.75)	151 (12.66)	70 (15.91)	245 (11.15)	40.87	<0.001
<1 y schooling	15 979 (73.09)	12 926 (71.69)	896 (75.10)	357 (81.14)	1800 (81.89)	129.86	<0.001
Current married	3536 (16.17)	3100 (17.19)	175 (14.67)	63 (14.32)	198 (9.01)	113.14	<0.001
BMI, kg/m^2,^ x̄ (SD)	18.99 ± 3.53	19.06 ± 3.51	19.12 ± 3.50	18.89 ± 3.62	18.33 ± 3.63	27.76	<0.001
Smoking	3177 (14.53)	2833 (15.71)	135 (11.32)	50 (11.36)	159 (7.23)	146.54	<0.001
Alcohol consumption	3900 (17.84)	3453 (19.15)	172 (14.42)	58 (13.18)	217 (9.87)	147.78	<0.001
Regular exercise	5237 (23.96)	4738 (26.28)	235 (19.70)	68 (15.45)	196 (8.92)	419.06	<0.001
ADL impairment	7688 (35.17)	5187 (28.77)	617 (51.72)	261 (59.32)	1623 (73.84)	1940.79	<0.001
Cognitive impairment	6753 (30.89)	5045 (27.98)	504(42.25)	149(33.86)	1055 (48.00)	446.71	<0.001
Hypertension	3346 (15.31)	2674 (14.83)	235 (19.70)	89 (20.23)	348 (15.83)	27.77	<0.001
Diabetes	310 (1.42)	224 (1.24)	28 (2.35)	10 (2.27)	48 (2.18)	20.18	<0.001
Heart disease	1559 (7.13)	1230 (6.82)	113 (9.47)	43 (9.77)	173 (7.87)	17.59	0.001
Cerebrovascular disease	1064(4.87)	630 (3.49)	123 (10.31)	72 (16.36)	239(10.87)	348.16	<0.001
Respiratory disease	2493 (11.40)	2006 (11.13)	152 (12.74)	49 (11.14)	286 (13.01)	8.87	0.031

ADL – activities of daily living, BMI – body mass index, SD – standard deviation, x̄ – mean

*Presented as n (%) unless specified otherwise.

†*P*-values were generated using variance analyses or χ^2^ test.

### Survival outcomes

**Figure 2:**
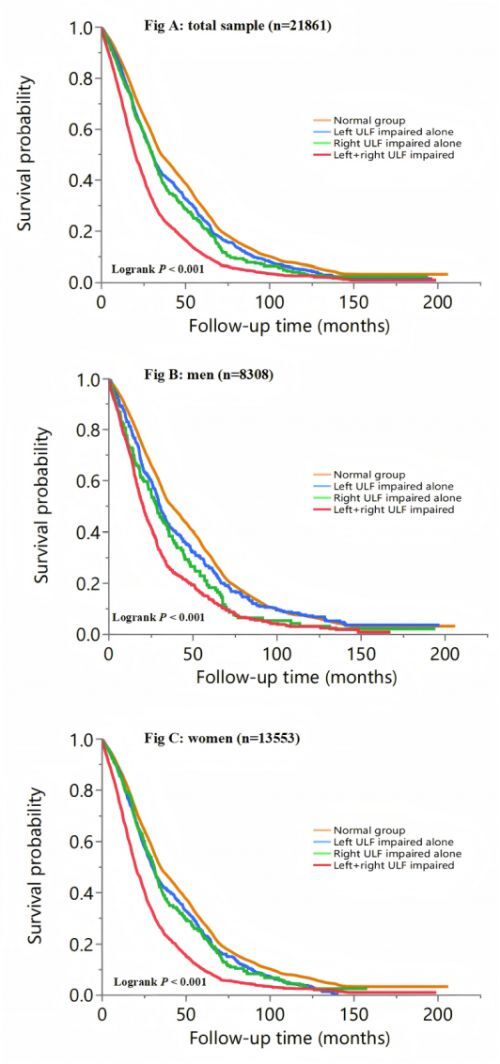
Kaplan-Meier survival curves for 21 861 participants and gender subgroups stratified by upper limb flexibility.

A total of 18 570 individuals died after 72 586.42 person-years of follow-up. The mortality density was highest in the group with bilateral ULF impairment, at 416.95 per 1000 person-years. The log-rank test showed significant differences (*P* < 0.001) in survival probabilities among participants and gender subgroups across different ULF groups (**Figure 2**).

### Independent association of left and right ULF impairment with mortality risk

In the overall sample, both left ULF impairment (HR = 1.06; 95% CI = 1.01, 1.12) and right ULF impairment (HR = 1.12; 95% CI = 1.05, 1.20) were significantly associated with higher all-cause mortality after accounting for all relevant covariates (**Table 2**). Gender-stratified subgroup analyses revealed that the association between left ULF impairment and mortality was more pronounced in women (HR = 1.09; 95% CI = 1.02, 1.16, *P* = 0.009) than in men (HR = 1.01; 95% CI = 0.92, 1.10, *P* = 0.748), with a statistically significant interaction (*P* = 0.014). Similarly, a significant interaction between right ULF impairment and gender was observed (*P* = 0.026).

**Table 2:** Independent associations of left and right ULF impairment with all-cause mortality risk

Group	Participant	Death	Person years	Model 1*		Model 2†		Model 3‡	
				**HR (95% CI)**	***P*-value**	**HR (95% CI)**	***P*-value**	**HR (95% CI)**	***P*-value**
**Left limb**									
Total sample									
*ULF normal*	18 470	15 478	63 888.18	Ref.		Ref.		Ref.	
*ULF impaired*	3391	3092	8698.24	1.33 (1.28, 1.38)	<0.001	1.28 (1.23, 1.33)	<0.001	1.06 (1.01, 1.12)	0.038
Men									
*ULF normal*	7342	6150	25 843.84	Ref.		Ref.		Ref.	
*ULF impaired*	966	869	2627.52	1.26 (1.17, 1.36)	<0.001	1.19 (1.11, 1.29)	<0.001	1.01 (0.92, 1.10)	0.748
Women									
*ULF normal*	11 128	9328	38 057.76	Ref.		Ref.		Ref.	
*ULF impaired*	2425	2223	6064.93	1.36 (1.29, 1.42)	<0.001	1.32 (1.26, 1.38)	<0.001	1.09 (1.02, 1.16)	0.009
**Right limb**									
Total sample									
*ULF normal*	19 223	16 132	66 357.82	Ref.		Ref.		Ref.	
*ULF impaired*	2638	2438	6228.6	1.45 (1.39, 1.51)	<0.001	1.38 (1.32, 1.44)	<0.001	1.12 (1.05, 1.20)	<0.001
Men									
*ULF normal*	7620	6388	26 822.41	Ref.		Ref.		Ref.	
*ULF impaired*	688	631	1658.08	1.43 (1.32, 1.56)	<0.001	1.34 (1.23, 1.46)	<0.001	1.08 (0.96, 1.23)	0.184
Women									
*ULF normal*	11 603	9744	39 531.42	Ref.		Ref.		Ref.	
*ULF impaired*	1950	1807	4568.85	1.45 (1.38, 1.53)	<0.001	1.39 (1.32, 1.47)	<0.001	1.14 (1.05, 1.23)	0.001

BMI – body mass index, CI – confidence interval, HR – hazard ratio, ref – reference, ULF – upper limb flexibility

*Model 1: adjusted for age and sex (not in sex-subgroup analyses).

†Model 2: additionally adjusted for ethnicity, residence, living arrangement, education, marital status, smoking, alcohol consumption, regular exercise, and BMI.

‡Model 3: additionally adjusted for ADL impairment, cognitive function, hypertension, diabetes, heart disease, cerebrovascular disease, and respiratory disease.

**Figure 3:**
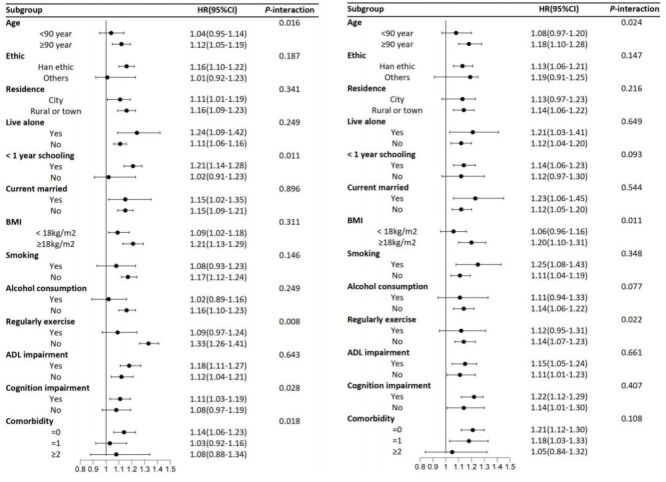
Subgroup analysis of left and right ULF impairment with all-cause mortality. All analyses were adjusted for age, sex, ethnicity, residence, living arrangement, education, marital status, smoking, alcohol consumption, regular exercise, BMI, ADL impairment, cognitive function, hypertension, diabetes, heart disease, cerebrovascular disease, and respiratory disease. **Panel A**. Subgroup analyses of left ULF impairment. **Panel B**. Subgroup analyses of right ULF impairment. ADL – activities of daily living, BMI – body mass index, ULF – upper limb flexibility.

In addition, we conducted subgroup analyses for left ULF impairment and right ULF impairment, respectively. The association between left ULF impairment and mortality was more pronounced in participants age ≥90 years, had no regular exercise habit, had cognitive impairment (**Figure 3****,** Panel A). In the subgroup analyses of right ULF, we found that age, BMI, alcohol consumption, and regular exercise significantly modified the relationship between right ULF and all-cause mortality (all *P*-interaction <0.05) (**Figure 3**, Panel B). Notably, the effect of left or right ULF impairment on mortality risk was not influenced by comorbidity (**Figure 3**).

### Synergetic effect of left and right ULF impaired on mortality risk

Participants with bilateral ULF impairment had the highest mortality risk (HR = 1.29; 95% CI = 1.19, 1.38) compared to the normal group, exceeding the risk for those with only left ULF impairment (HR = 1.06; 95% CI = 0.98, 1.14) or only right ULF impairment (HR = 1.13; 95% CI = 1.01, 1.31). This trend was consistent across both genders. In the total sample, the RERI = 0.10 (95% CI = 0.02, 0.18) and the SI = 1.52 (95% CI = 1.13, 2.06), indicating a significant additive effect of combined left and right ULF impairments on mortality risk. The proportion of mortality risk attributable to the interaction effect was 8% (AP = 0.08; 95% CI = 0.05, 0.11). Subgroup analyses by gender showed that the synergistic effect was more pronounced in women (**Table 3**).

**Table 3:** Additive interactions between left and right ULF impairment on all-cause mortality*

Group	Participant	Death	Person years	Adjusted HR (95% CI)	*P*-value	RERI (95% CI)	AP (95% CI)	SI (95% CI)
**Overall (n = 21 861)**						0.10 (0.02, 0.18)	0.08 (0.05–0.11)	1.52 (1.13, 2.06)
Normal	18 030	15 086	62 566.64					
Left impaired alone	1193	1046	3791.18	1.06 (0.98, 1.14)	0.116			
Right impaired alone	440	392	1321.53	1.13 (1.01, 1.31)	0.035			
Left + right impaired	2198	2046	4907.06	1.29 (1.19, 1.38)	<0.001			
**Men (n = 8308)**						0.01 (−0.07, 0.09)	0.01 (−0.02, 0.04)	1.04 (0.76, 1.43)
Normal	7213	6033	25 476.46					
Left impaired alone	407	355	1343.53	1.03 (0.91, 1.16)	0.656			
Right impaired alone	129	117	369.33	1.21 (1.02, 1.43)	0.031			
Left + right impaired	559	514	1289.63	1.25 (1.06, 1.52)	0.003			
**Women (n = 103 553)**						0.13 (0.05, 0.21)	0.09 (0.07, 0.13)	1.72 (1.26, 2.34)
Normal	10817	9053	37 090.18					
Left impaired alone	786	691	2447.65	1.08 (0.98, 1.18)	0.101			
Right impaired alone	311	275	952.2	1.10 (0.94, 1.28)	0.228			
Left + right impaired	1639	1532	3617.44	1.31 (1.20, 1.42)	<0.001			

AP – attributable proportion due to interaction, CI – confidence interval, HR – hazard ratio, RERI – relative excess risk due to interaction, SI – synergy index, ULF – upper limb flexibility

*Adjusted for age, sex, ethnicity, residence, living arrangement, education, marital status, smoking, alcohol consumption, regular exercise, body mass index, activities of daily living impairment, cognitive function, hypertension, diabetes, heart disease, cerebrovascular disease, and respiratory disease.

### Sensitivity analysis

The core findings demonstrated substantial stability throughout the sensitivity analyses. After excluding participants who died in the first year or those with a history of heart or cerebrovascular disease, the independent associations of ULF impairment with all-cause mortality remained statistically significant (Table S2–4 in the **Online Supplementary Document**). The synergistic effect between left and right ULF impairments was also confirmed, with RERIs consistently greater than zero (Table S5–7 in the **Online Supplementary Document**).

After excluding individuals with severe cognitive impairment, right ULF impairment remained significantly associated with mortality (adjusted HR = 1.12; 95% CI = 1.05, 1.20, *P* = 0.001), and the association between left ULF impairment and mortality was no longer statistically significant (adjusted HR = 1.05, *P* = 0.061) (Table S5 in the **Online Supplementary Document**). Similarly, after excluding participants with severe physical impairment, the association between right ULF impairment and mortality remained significant (adjusted HR = 1.11, *P* = 0.008). Conversely, the association between left ULF impairment and mortality did not reach statistical significance (adjusted HR = 1.05, *P* = 0.134) (Table S9 in the **Online Supplementary Document**). Besides, there were no significant change in the additive interaction between left ULF impairment and right ULF impairment on all-cause mortality when participants with severe cognitive impairment or severe physical impairment were excluded (Table S10–11 in the **Online Supplementary Document**).

## DISCUSSION

In this prospective cohort study, left ULF impairment and right ULF impairment were both associated with higher all-cause mortality. In the current study, individuals with ULF impairment account for 17.52% of the total sample, highlighting the necessity of screening for limb flexibility in the oldest-old population. In the older population, ULF is closely related to overall balance ability. High flexibility of the upper limbs can enhance the sensory input of the oldest-old, including visual, vestibular, and proprioceptive inputs, which are key factors in maintaining balance. Reduced balance ability increases the risk of falls in the older adults [34]. The Functional Reach Test (FRT), developed by Duncan et al. in 1990, assesses balance function by evaluating participants' ability to extend their upper limbs horizontally forward [35]. Li Fengxia et al demonstrated a high correlation between the range of forward and backward arm movement in FRT testing and the incidence of falls [36]. In comparison with the FRT, our suite of testing indicators offers greater diversity. They assess not only the horizontal flexibility of the upper limbs among the oldest-old population but also incorporate various directions and angles. This comprehensive approach could potentially serve as a valuable tool for predicting fall risk in individuals.

Independent analysis indicated that left-sided ULF impairment was associated with a 6% increased risk of death, and right-sided ULF impairment is associated with a 12% increased risk of death. In the subgroup analysis, older individuals aged ≥90 years exhibited an increased risk of bilateral ULF impairment, with the right-sided impairment risk being more pronounced. These results indicated that the risk of right-sided ULF impairment on mortality was greater than that of left-sided ULF impairment. The right hand, often the dominant one, compared to the non-dominant hand, when injured, can significantly impact the daily living abilities of the older adults, causing a loss of independence and an increase in dependence on external assistance [37,38]. This loss of self-care ability may lead to an overall deterioration of physical health, thereby increasing the risk of death [39].

The study also identified a synergistic effect of left and right ULF impairment on mortality, indicating that the risk of death due to bilateral ULF impairment is significantly higher than that due to unilateral ULF impairment. The human body relies on coordinated bilateral motor control for balance and daily activities [38,40]. When both sides of the upper limbs are impaired, it can lead to a more significant disruption in balance and coordination than unilateral impairment alone. In cases of unilateral ULF impairment, the unaffected side can often compensate for the loss of function, providing some level of adaptation and maintaining daily activities. However, when both sides are impaired, these compensatory mechanisms are overwhelmed, leading to a higher risk of falls and reduced ability to perform daily tasks [41,42].

In the gender subgroup analysis, a more pronounced association was observed between ULF impairment and mortality among the female oldest-old population. Research has found that among the oldest-old and long-lived individuals, although women have longer lifespans, their health status is relatively poorer, and they are more sensitive to reduced physical function [43,44]. Women typically exhibit lower muscle mass and strength compared to men, which can negatively impact their ULF and overall functional capacity. With advancing age, there is a decline in muscle mass and strength, which can render daily activities more challenging for them, thereby elevating the risk of mortality. Women's physical activity levels are generally lower than men [45]. This difference may make women more susceptible to decreased ULF in old age. Lack of physical activity accelerates muscle loss and reduces overall body function, thereby increasing the risk of death. Women often act as caregivers and take on more household chores and other labour, which can lead to increased physical strain and a higher incidence of hand disorders, thereby affecting ULF and potentially mortality [46]. In addition, there may be differences between women and men in reporting their own functional abilities. Women may be more likely to report physical discomfort or functional limitations, which may lead to stronger correlations observed in the study.

As shown in **Table 1**, ULF is significantly correlated with daily activity ability, cognitive function, and various chronic diseases among the older adults. Furthermore, in the multivariate Cox regression analysis, the HR value was reduced after adjusting for health status factors. This evidence suggests that the relationship between ULF and mortality can be explained by ADL, cognitive function, and chronic diseases. The term ADL denotes the capacity of an individual to autonomously manage fundamental daily activities. Zhiwen Ge et al found that older individuals with a decline in ADL have a higher mortality rate than those without such a decline [47]. Jimbo et al. found significant differences in upper-limb function and ADL among patients after cervical spinal cord injury [48]. The association between impaired ULF and elevated mortality in the oldest-old may operate through its adverse effects on ADL capacity. Kazuo et al.'s study demonstrated that participants who did not partaking in outdoor activities had nearly double the mortality risk almost twice that of participants indulging in them [49]. Older adults with ULF impairment are likely to have reduced participation in outdoor and leisure activities owing to a decreased ability to engage in daily activities, which can lead to an increased mortality rate.

Cognitive function may also contribute to the association between ULF and mortality. Lv et al. found that the faster the cognitive decline in older people, the higher the mortality rate, but this is not related to the initial level of cognitive function [50]. A scoping review has indicated that motor function variables of the upper limbs are typically correlated with cognitive impairments [51]. This correlation can be instrumental in differentiating cognitive impairment from the normal cognitive ageing process. These findings indicate that the impaired ULF in the oldest-old population may serve as a novel biomarker for cognitive impairment.

Upper limb flexibility in the oldest-old demographic is significantly associated with chronic conditions, including sarcopenia and frailty. A study from South Korean revealed that sarcopenia and sarcopenic obesity significantly adversely affect older adults' health and are strongly associated with various chronic diseases [52]. The Hisayama study from Japan also found that diabetes in the older adults is associated with the risk of sarcopenia [53]. These chronic conditions are intricately linked to key health outcomes in the older adults, such as quality of life, physical functionality, and the propensity for falls, which may lead to increased mortality.

In this study, 99.69% of the oldest-old successfully completed the measurement. Compared to current physical function assessments, this index offers the advantage of being simple and easy to implement, requiring no professional medical guidance or equipment, and can be administered independently, especially in research among the oldest-old. It is also applicable to older individuals with walking difficulties or limited ADL, and its convenience and user-friendly nature make it ideal for home-based assessment. Traditional ADL assessment tools, such as the Barthel Index and Katz Index, primarily focus on whether individuals can complete activities independently and the extent of assistance required. However, these scales frequently overlook the quality of task execution, particularly the role of ULF. Incorporating ULF into ADL assessments can provide a more comprehensive reflection of upper limb function in the older adults and aid in identifying potential risk factors. Furthermore, this research, grounded in a nationally representative sample of China's older population, offers a distinctive chance to gauge the correlation between the ULF and all-cause mortality.

Several limitations of this study should also be acknowledged. First, the study population was primarily limited to Chinese older individuals, lacking representation from other countries and regions. Future research should incorporate more diverse samples to enhance generalisability. Second, like other observational research, it is not feasible to eliminate the impact of all potential confounding variables such as genetic risk and environmental factors. Third, since ULF was assessed only at a single time point in this study, longitudinal evaluations should be considered in future research to account for potential temporal variations. Fourth, due to data limitations, our outcome only analysed all-cause mortality and did not analyse specific causes of death. And our study did not speculate on potential mediating factors. Furthermore, we assessed chronic diseases in the older adults exclusively via self-report questionnaires, which may introduce recall bias and compromise measurement accuracy. Future research should consider diagnosis of different diseases, especially neurodegenerative disease. This study may be subject to attrition bias, as some participants were lost to follow-up due to advanced age or deteriorating health status. While our study benefits from high statistical power due to substantial mortality events and extended follow-up, the focus on the older adults raises potential concerns regarding reverse causality. However, sensitivity analyses excluding participants with extremely old individuals, early deaths, and severe health issues yielded consistent results. Future longitudinal studies incorporating repeated ULF assessments would help clarify causal relationships.

## CONCLUSIONS

Impaired ULF is associated with a higher risk of mortality, especially when the impairment occurs on the right side. For the oldest-old, ULF may serve as a simple and effective predictor of premature death.

## Additional material


Online Supplementary Document

## References

[1] Milanović Z Pantelić S Trajković N Sporiš G Kostić R James N Age-related decrease in physical activity and functional fitness among elderly men and women. Clin Interv Aging. 2013;8:549–56. 10.2147/CIA.S44112 23723694 PMC3665513

[2] Tu W-J Zeng X Liu Q Aging tsunami coming: the main finding from China’s seventh national population census. Aging Clin Exp Res. 2022;34:1159–63. 10.1007/s40520-021-02017-4 34727357

[3] Jiménez-García JD Martínez-Amat A Hita-Contreras F Fábrega-Cuadros R Álvarez-Salvago F Aibar-Almazán A Muscle Strength and Physical Performance Are Associated with Reaction Time Performance in Older People. Int J Environ Res Public Health. 2021;18:5893. 10.3390/ijerph18115893 34072660 PMC8197826

[4] Zadik S Benady A Gutwillig S Florentine MM Solymani RE Plotnik M Age related changes in gait variability, asymmetry, and bilateral coordination - When does deterioration starts? Gait Posture. 2022;96:87–92. 10.1016/j.gaitpost.2022.05.009 35617787

[5] Reimann H Ramadan R Fettrow T Hafer JF Geyer H Jeka JJ Interactions Between Different Age-Related Factors Affecting Balance Control in Walking. Front Sports Act Living. 2020;2:94. 10.3389/fspor.2020.00094 33345085 PMC7739654

[6] Portegijs E Buurman BM Essink-Bot ML Zwinderman AH de Rooij SE Failure to regain function at 3 months after acute hospital admission predicts institutionalization within 12 months in older patients. J Am Med Dir Assoc. 2012;13:569.e1–7. 10.1016/j.jamda.2012.04.003 22572555

[7] Pereira CLN Vogelaere P Baptista F Role of physical activity in the prevention of falls and their consequences in the elderly. Eur Rev Aging Phys Act. 2008;5:51–8. 10.1007/s11556-008-0031-8

[8] Paterson DH Warburton DER Physical activity and functional limitations in older adults: a systematic review related to Canada’s Physical Activity Guidelines. Int J Behav Nutr Phys Act. 2010;7:38. 10.1186/1479-5868-7-38 20459782 PMC2882898

[9] Sánchez-Sánchez JL Lu W-H Gallardo-Gómez D del Pozo Cruz B de Souto Barreto P Lucia A Association of intrinsic capacity with functional decline and mortality in older adults: a systematic review and meta-analysis of longitudinal studies. Lancet Healthy Longev. 2024;5:e480–92. 10.1016/S2666-7568(24)00092-8 38945130

[10] Patrizio E Calvani R Marzetti E Cesari M Physical Functional Assessment in Older Adults. J Frailty Aging. 2021;10:141–9. 10.14283/jfa.2020.61 33575703

[11] PereraSZhangXPattersonCGBoudreauRMSerial gait speed measurements over time and dynamic survival prediction in older adults. J Nutr Health Aging. 2024;28:100330. 10.1016/j.jnha.2024.10033039128300

[12] Núñez-Cortés R Cruz BDP Gallardo-Gómez D Calatayud J Cruz-Montecinos C López-Gil JF Handgrip strength measurement protocols for all-cause and cause-specific mortality outcomes in more than 3 million participants: A systematic review and meta-regression analysis. Clin Nutr. 2022;41:2473–89. 10.1016/j.clnu.2022.09.006 36215867

[13] Hsu NW Lin CH Yang NP Chen HC Chou P Handgrip strength is associated with mortality in community-dwelling older adults: the Yilan cohort study, Taiwan. BMC Public Health. 2023;23:2194. 10.1186/s12889-023-17058-9 37940899 PMC10631044

[14] Xie K Han X Hu X Balance ability and all-cause death in middle-aged and older adults: A prospective cohort study. Front Public Health. 2023;10:1039522. 10.3389/fpubh.2022.1039522 36699907 PMC9868834

[15] Jason MRG. Physical activity and mortality. In Stensel DJ, Hardman AE, Gill JMR, editors. Physical Activity and Health. London, UK: Routledge; 2021. p. 63-95.

[16] Chen LK Woo J Assantachai P Auyeung TW Chou MY Iijima K Asian Working Group for Sarcopenia: 2019 Consensus Update on Sarcopenia Diagnosis and Treatment. J Am Med Dir Assoc. 2020;21:300–307.e2. 10.1016/j.jamda.2019.12.012 32033882

[17] Franzke B Neubauer O Cameron-Smith D Wagner KH Dietary Protein, Muscle and Physical Function in the Very Old. Nutrients. 2018;10:935. 10.3390/nu10070935 30037048 PMC6073115

[18] Cruz-Jentoft AJ Bahat G Bauer J Boirie Y Bruyère O Cederholm T Sarcopenia: revised European consensus on definition and diagnosis. Age Ageing. 2019;48:16–31. 10.1093/ageing/afy169 30312372 PMC6322506

[19] Freiberger E de Vreede P Schoene D Rydwik E Mueller V Frändin K Performance-based physical function in older community-dwelling persons: a systematic review of instruments. Age Ageing. 2012;41:712–21. 10.1093/ageing/afs099 22885845

[20] Pavasini R Guralnik J Brown JC di Bari M Cesari M Landi F Short Physical Performance Battery and all-cause mortality: systematic review and meta-analysis. BMC Med. 2016;14:215. 10.1186/s12916-016-0763-7 28003033 PMC5178082

[21] Lee H Lee E Jang IY Frailty and Comprehensive Geriatric Assessment. J Korean Med Sci. 2020;35:e16. 10.3346/jkms.2020.35.e16 31950775 PMC6970074

[22] Khanal P He L Stebbings GK Onambele-Pearson GL Degens H Williams AG Static one-leg standing balance test as a screening tool for low muscle mass in healthy elderly women. Aging Clin Exp Res. 2021;33:1831–9. 10.1007/s40520-021-01818-x 33715139 PMC8249245

[23] Schultz AB Mobility impairment in the elderly: challenges for biomechanics research. J Biomech. 1992;25:519–28. 10.1016/0021-9290(92)90092-F 1592857

[24] Berme N, Heydinger G, Engin AE. Biomechanics of the Joints in the Upper Limb. In Berme N, Engin AE, Correia da Silva KM, editors. Biomechanics of Normal and Pathological Human Articulating Joints. Dordrecht, the Netherlands: Springer; 1985. p. 115-135.

[25] Pei D, Burns M, Chandramouli R, Vinjamuri R. Neural Decoding of Upper Limb Movements Using Electroencephalography. In Guger C, Allison BZ, Miller K, editors. Brain–Computer Interface Research. SpringerBriefs in Electrical and Computer Engineering. Cham, Switzerland: Springer; 2020. p. 25-33.

[26] Owada H Otomo A Suzuki Y Suto A Murakami K Kishikawa Y The relationship between frailty and motor function among living in the community elderly females. J Phys Ther Sci. 2023;35:70–4. 10.1589/jpts.35.70 36628139 PMC9822822

[27] Letícia Prieto T Alessandra Vidal P Rafael Marcos Dias C Michelly Rodrigues C Inácio Carlos Murta J Renata Nunes L Correlation between sarcopenia, risk of falls and mortality in the elderly - systematic review and meta-analysis of observational studies. Arq Ciênc Saúde UNIPAR. 2023;27:5704–21.

[28] Atroshi I Gummesson C Andersson B Dahlgren E Johansson A The disabilities of the arm, shoulder and hand (DASH) outcome questionnaire: reliability and validity of the Swedish version evaluated in 176 patients. Acta Orthop Scand. 2000;71:613–8. 10.1080/000164700317362262 11145390

[29] David PG Jolie R Jonathan CI Ralph G Patrick G Dale B Assessment of the Disabilities of the Arm, Shoulder, and Hand (DASH) questionnaire for use in patients after neck dissection for head and neck cancer. Head Neck. 2014;37:234–42. 24375871 10.1002/hed.23593

[30] Jester A Harth A Wind G Germann G Sauerbier M Disabilities of the Arm, Shoulder and Hand (Dash) Questionnaire: Determining Functional Activity Profiles in Patients with Upper Extremity Disorders. J Hand Surg Br. 2005;30:23–8. 10.1016/J.JHSB.2004.08.008 15620487

[31] Huang X Zhang M Fang J Growth patterns of activity of daily living disability and associated factors among the Chinese elderly: A twelve-year longitudinal study. Arch Gerontol Geriatr. 2022;99:104599. 10.1016/j.archger.2021.104599 34902707

[32] Katzman R Zhang MY Ouang Ya Q Wang ZY Liu WT Yu E A Chinese version of the Mini-Mental State Examination; impact of illiteracy in a Shanghai dementia survey. J Clin Epidemiol. 1988;41:971–8. 10.1016/0895-4356(88)90034-0 3193141

[33] Tombaugh TN McIntyre NJ The mini-mental state examination: a comprehensive review. J Am Geriatr Soc. 1992;40:922–35. 10.1111/j.1532-5415.1992.tb01992.x 1512391

[34] Xing L Bao Y Wang B Shi M Wei Y Huang X Falls caused by balance disorders in the elderly with multiple systems involved: Pathogenic mechanisms and treatment strategies. Front Neurol. 2023;14:1128092. 10.3389/fneur.2023.1128092 36908603 PMC9996061

[35] Duncan PW Weiner DK Chandler J Studenski S Functional reach: a new clinical measure of balance. J Gerontol. 1990;45:M192–7. 10.1093/geronj/45.6.M192 2229941

[36] Dong-Mei LF-XWXC Application of Functional Reach Test in Assessment of Fall Risk in Stroke Patients with Hemiplegia. Chinese Journal of Rehabilitation Theory and Practice. 2010;16:969–72.

[37] Kalisch T Wilimzig C Kleibel N Tegenthoff M Dinse HR Age-related attenuation of dominant hand superiority. PLoS One. 2006;1:e90. 10.1371/journal.pone.0000090 17183722 PMC1762407

[38] Kilbreath SL Heard RC Frequency of hand use in healthy older persons. Aust J Physiother. 2005;51:119–22. 10.1016/S0004-9514(05)70040-4 15924514

[39] Tamulevicius M Bucher F Dastagir N Maerz V Vogt PM Dastagir K Demographic shifts reshaping the landscape of hand trauma: a comprehensive single-center analysis of changing trends in hand injuries from 2007 to 2022. Inj Epidemiol. 2024;11:25. 10.1186/s40621-024-00510-8 38872185 PMC11170831

[40] Bailey RR Klaesner JW Lang CE Quantifying Real-World Upper-Limb Activity in Nondisabled Adults and Adults With Chronic Stroke. Neurorehabil Neural Repair. 2015;29:969–78. 10.1177/1545968315583720 25896988 PMC4615281

[41] Shirota C Jansa J Diaz J Balasubramanian S Mazzoleni S Borghese NA On the assessment of coordination between upper extremities: towards a common language between rehabilitation engineers, clinicians and neuroscientists. J Neuroeng Rehabil. 2016;13:80. 10.1186/s12984-016-0186-x 27608923 PMC5017057

[42] Shih PC Steele CJ Hoepfel D Muffel T Villringer A Sehm B The impact of lesion side on bilateral upper limb coordination after stroke. J Neuroeng Rehabil. 2023;20:166. 10.1186/s12984-023-01288-4 38093308 PMC10717693

[43] Farrelly C Longevity Science and Women’s Health and Wellbeing. J Popul Ageing. 2023;18:1–20. 36741335 10.1007/s12062-023-09411-y PMC9885070

[44] Vega-Alonso T Lozano-Alonso J Estévez-Iglesias L Ordax-Díez A Arrieta-Antón E Díaz-Rodríguez Á Health and wellbeing status of the long-lived individuals of the Spanish LONGECYL cross-sectional study. Arch Public Health. 2024;82:77. 10.1186/s13690-024-01305-5 38769585 PMC11103821

[45] Young-Shin L Gender Differences in Physical Activity and Walking Among Older Adults. Journal of Women &amp. Aging. 2005;17:55–70. 10.1300/J074v17n01_05 15914419

[46] Leow MQH Teo W Low TL Tay SC Hand Assessment for Elderly People in the Community. Orthop Nurs. 2019;38:25–30. 10.1097/NOR.0000000000000515 30676573

[47] Ge Z Li C Li Y Wang N Hong Z Lifestyle and ADL Are Prioritized Factors Influencing All-Cause Mortality Risk Among Oldest Old: A Population-Based Cohort Study. Inquiry. 2024;61:469580241235755. 10.1177/00469580241235755 38411099 PMC10901056

[48] Jimbo K Miyata K Yuine H Takahama K Yoshimura T Shiba H Classification of upper-limb dysfunction severity and prediction of independence in activities of daily living after cervical spinal-cord injury. Spinal Cord. 2024;62:507–13. 10.1038/s41393-024-01005-5 38886575

[49] Inoue K Shono T Matsumoto M Absence of outdoor activity and mortality risk in older adults living at home. J Aging Phys Act. 2006;14:203–11. 10.1123/japa.14.2.203 19462550

[50] Lv X Li W Ma Y Chen H Zeng Y Yu X Cognitive decline and mortality among community-dwelling Chinese older people. BMC Med. 2019;17:63. 10.1186/s12916-019-1295-8 30871536 PMC6419492

[51] Rudd KD Lawler K Callisaya ML Alty J Investigating the associations between upper limb motor function and cognitive impairment: a scoping review. Geroscience. 2023;45:3449–73. 10.1007/s11357-023-00844-z 37337026 PMC10643613

[52] Lim HS Park YH Suh K Yoo MH Park HK Kim HJ Association between Sarcopenia, Sarcopenic Obesity, and Chronic Disease in Korean Elderly. J Bone Metab. 2018;25:187. 10.11005/jbm.2018.25.3.187 30237999 PMC6135652

[53] Nakamura K Yoshida D Honda T Hata J Shibata M Hirakawa Y Midlife and late-life diabetes and sarcopenia in a general older Japanese population: The Hisayama Study. J Diabetes Investig. 2021;12:1899–907. 10.1111/jdi.13550 33742564 PMC8504915

