# Supplementary material for: Associations between upper limb flexibility and all-cause mortality in the oldest-old
Source: J Glob Health. 2025 Oct 3;15:04224. doi: 10.7189/jogh.15.04224 (PMC12491906; doi:10.7189/jogh.15.04224)
Supplement: Online Supplementary Document [file jogh-15-04224-s001.pdf]

Supplement to: Zhang Y, Zhang C, Hu J, Kang Y, Zhang J, Zhao J, Shi H, Shen J. Associations between upper limb flexibility and all-cause mortality in the oldest-old. J Glob Health. 2025;15:04224.

Supplementary table 1. Comparison of characteristics between the samples finally included in the analysis and the samples lost to follow-up

Characteristics	Included sample	Lost to follow up	t/x^2	*P* -value
Participants, n	21861	4164		
Age, year	92.61±7.23	93.75±7.16	-7.21	<0.001
Female, n (%)	13553 (62.00)	2596(62.34)	0.18	0.671
Han ethnic, n (%)	20442 (93.51)	4054(97.36)	112.53	<0.001
Rural or town, n (%)	17704 (80.98)	2675(64.24)	524.36	<0.001
Live alone, n (%)	3305 (15.12)	638(15.32)	0.112	0.737
< 1 year schooling, n (%)	15979 (73.09)	2816(67.63)	25.44	<0.001
Current married, n (%)	3536 (16.17)	584(14.02)	12.45	<0.001
BMI, kg/m^2	18.99±3.53	19.45±3.59	-7.65	<0.001
Smoking, n (%)	3177 (14.53)	514(12.34)	14.21	<0.001
Alcohol consumption, n (%)	3900 (17.84)	516(12.39)	78.71	<0.001
Regular exercise, n (%)	5237 (23.96)	954(22.91)	2.11	0.146
ADL impairment, n (%)	7688(35.17)	1766(42.41)	78.02	<0.001
Cognitive impairment, n (%)	6753 (30.89)	1546(37.13)	62.64	<0.001
Hypertension, n (%)	3346 (15.31)	693(16.64)	4.69	0.031
Diabetes, n (%)	310 (1.42)	114(2.74)	32.91	<0.001
Heart disease, n (%)	1559 (7.13)	391(9.39)	24.31	<0.001
Cerebrovascular disease, n (%)	1064(4.87)	244(5.86)	6.93	0.008
Respiratory disease, n (%)	2493 (11.40)	471(11.31)	0.031	0.863

Supplementary table 2. Independent associations of left and right ULF impairment with mortality risk after excluding 2324 descents in the first year

19537 participants were included

Group	Participant	Death	Person years	Fully adjusted model	
				HR (95% CI)	*P*-value
**Left limb**					
ULF normal	16728	13736	63207.29		
ULF impaired	2809	2510	8487.46	1.07(1.01-1.13)	0.028
**Right limb**					
ULF normal	17405	14314	65649.1		
ULF impaired	2132	1932	6045.65	1.13(1.05-1.21)	<0.001

Adjusted for age, sex, ethnicity, residence, living arrangement, education, marital status, smoking, alcohol consumption, regular exercise, BMI, ADL impairment, cognitive function, hypertension, diabetes, heart disease, cerebrovascular disease, and respiratory disease.
ULF, Upper limb flexibility; HR, hazard ratios; CI, confidence interval.

Supplementary table 3. Independent associations of left and right ULF impairment with mortality risk after excluding 1559 participants with heart disease

20302 participants were included

Group	Participant	Death	Person years	Fully adjusted model	
				HR (95% CI)	*P*-value
**Left limb**					
ULF normal	17197	14427	59556.57		
ULF impaired	3105	2834	7938.75	1.07(1.01-1.13)	0.025
**Right limb**					
ULF normal	17880	15028	61792.69		
ULF impaired	2422	2233	5702.63	1.14（1.06-1.21）	<0.001

Adjusted for age, sex, ethnicity, residence, living arrangement, education, marital status, smoking, alcohol consumption, regular exercise, BMI, ADL impairment, cognitive function, hypertension, diabetes, heart disease, cerebrovascular disease, and respiratory disease.
ULF, Upper limb flexibility; HR, hazard ratios; CI, confidence interval.

Supplementary table 4. Independent associations of left and right ULF impairment with mortality risk after excluding 1064 participants with cerebrovascular disease

20797 participants were included

Group	Participant	Death	Person years	Fully adjusted model	
				HR (95% CI)	*P*-value
**Left limb**					
ULF normal	17768	14919	61603.51		
ULF impaired	3029	2762	7896.45	1.06(1.00-1.12)	0.046
**Right limb**					
ULF normal	18470	15530	63930.79		
ULF impaired	2327	2151	5569.16	1.13(1.06-1.21)	<0.001

Adjusted for age, sex, ethnicity, residence, living arrangement, education, marital status, smoking, alcohol consumption, regular exercise, BMI, ADL impairment, cognitive function, hypertension, diabetes, heart disease, cerebrovascular disease, and respiratory disease.
ULF, Upper limb flexibility; HR, hazard ratios; CI, confidence interval.

Supplementary table 5. Additive interactions between left and right ULF impairment on all-cause mortality

after excluding 1559 participants with heart disease

20302 participants were included

**Group**	**Participant**	**Death**	**Person years**	**Adj-HR (95% CI)**	***P*-value**	**RERI (95%CI)**	**AP (95%CI)**	**SI (95%CI)**
**Overall (n = 20302)**						0.11 (0.03–0.19)	0.08 (0.06–0.11)	1.55 (1.16–2.06)
Normal	16800	1407	558376.5					
Left impaired alone	1080	953	3416.19	1.07 (0.99–1.15)	0.081			
Right impaired alone	397	352	1180.07	1.13 (1.00–1.19)	0.043			
Left + right impaired	2025	1881	4522.56	1.31 (1.21–1.40)	<0.001			

Adjusted for age, sex, ethnicity, residence, living arrangement, education, marital status, smoking, alcohol consumption, regular exercise, BMI, ADL impairment, cognitive function, hypertension, diabetes, heart disease, cerebrovascular disease, and respiratory disease.

ULF, Upper limb flexibility; HR, hazard ratios; CI, confidence interval; RERI, relative excess risk due to interaction; AP, attributable proportion due to interaction; SI, synergy index.

Supplementary table 6. Additive interactions between left and right ULF impairment on all-cause mortality

after excluding 1064 participants with cerebrovascular disease

20302 participants were included

Group	Participant	Death	Person years	Adj-HR (95% CI)	*P*-value	RERI (95%CI)	AP (95%CI)	SI (95%CI)
**Overall (n = 20302)**						0.14 (0.06–0.22)	0.11 (0.08–0.13)	1.88 (1.32–2.65)
Normal	17400	14593	60482.45					
Left impaired alone	1070	937	3448.34	1.04 (0.96–1.12)	0.298			
Right impaired alone	368	326	1121.06	1.12 (1.01–1.23)	0.032			
Left + right impaired	1959	1825	4448.1	1.30 (1.20–1.39)	<0.001			

Adjusted for age, sex, ethnicity, residence, living arrangement, education, marital status, smoking, alcohol consumption, regular exercise, BMI, ADL impairment, cognitive function, hypertension, diabetes, heart disease, cerebrovascular disease, and respiratory disease.
ULF, Upper limb flexibility; HR, hazard ratios; CI, confidence interval; RERI, relative excess risk due to interaction; AP, attributable proportion due to interaction; SI, synergy index.

Supplementary table 7. Additive interactions between left and right ULF impairment on all-cause mortality

after excluding 2324 descents in the first year

19537 participants were included

**Group**	**Participant**	**Death**	**Person years**	**Adj-HR (95% CI)**	***P*-value**	**RERI (95%CI)**	**AP (95%CI)**	**SI (95%CI)**
**Overall (n = 19537)**						0.13 (0.05–0.21)	0.10 (0.07–0.13)	1.86 (1.29–2.70)
Normal	16344	13400	61906.08					
Left impaired alone	1061	914	3743.02	1.04 (0.96–1.13)	0.271			
Right impaired alone	384	336	1301.21	1.11 (1.02–1.21)	0.027			
Left + right impaired	1748	1596	4744.44	1.28 (1.18–1.37)	<0.001			

Adjusted for age, sex, ethnicity, residence, living arrangement, education, marital status, smoking, alcohol consumption, regular exercise, BMI, ADL impairment, cognitive function, hypertension, diabetes, heart disease, cerebrovascular disease, and respiratory disease.

ULF, Upper limb flexibility; HR, hazard ratios; CI, confidence interval; RERI, relative excess risk due to interaction; AP, attributable proportion due to interaction; SI, synergy index.

Supplementary table 8. Independent associations of left and right ULF impairment with mortality risk after excluding 2753 participants with severe cognitive impairment

19108 participants were included

Group	Participant	Death	Person years	Fully adjusted model	
				HR (95% CI)	*P*-value
**Left limb**					
ULF normal	16328	13545	58200.49		
ULF impaired	2780	2519	7312.36	1.05(0.99-1.12)	0.061
**Right limb**					
ULF normal	16935	14062	60281.35		
ULF impaired	2173	2002	5231.51	1.12(1.05-1.20)	0.001

Adjusted for age, sex, ethnicity, residence, living arrangement, education, marital status, smoking, alcohol consumption, regular exercise, BMI, ADL impairment, cognitive function, hypertension, diabetes, heart disease, cerebrovascular disease, and respiratory disease.
ULF, Upper limb flexibility; HR, hazard ratios; CI, confidence interval.

Supplementary table 9. Independent associations of left and right ULF impairment with mortality risk after excluding 4601 participants with severe physical function impairment

17260 participants were included

Group	Participant	Death	Person years	Fully adjusted model	
				HR (95% CI)	*P*-value
**Left limb**					
ULF normal	15629	12807	57884.85		
ULF impaired	1631	1419	5466.82	1.05(0.98-1.11)	0.134
**Right limb**					
ULF normal	16151	13252	59824.94		
ULF impaired	1109	974	3526.73	1.11(1.03-1.19)	0.008

Adjusted for age, sex, ethnicity, residence, living arrangement, education, marital status, smoking, alcohol consumption, regular exercise, BMI, cognitive function, hypertension, diabetes, heart disease, cerebrovascular disease, and respiratory disease.
ULF, Upper limb flexibility; HR, hazard ratios; CI, confidence interval.

Supplementary table 10. Additive interactions between left and right ULF impairment on all-cause mortality after excluding 2753 participants with severe cognitive impairment

19108 participants were included

Group	Participant	Death	Person years	Adj-HR (95% CI)	*P*-value	RERI (95%CI)	AP (95%CI)	SI (95%CI)
**Overall (n = 19108)**						0.13 (0.05–0.21)	0.11 (0.08–0.13)	2.18 (1.30–3.65)
Normal	15949	13207	57031.80					
Left impaired alone	986	855	3249.55	1.03 (0.93–1.08)	0.271			
Right impaired alone	379	338	1168.69	1.09 (1.02–1.17)	0.019			
Left + right impaired	1794	1664	4062.82	1.24 (1.09–1.41)	<0.001			

Adjusted for age, sex, ethnicity, residence, living arrangement, education, marital status, smoking, alcohol consumption, regular exercise, BMI, ADL impairment, cognitive function, hypertension, diabetes, heart disease, cerebrovascular disease, and respiratory disease.

ULF, Upper limb flexibility; HR, hazard ratios; CI, confidence interval; RERI, relative excess risk due to interaction; AP, attributable proportion due to interaction; SI, synergy index.

Supplementary table 11. Additive interactions between left and right ULF impairment on all-cause mortality after excluding 4601 participants with severe physical function impairment

17260 participants were included

Group	Participant	Death	Person years	Adj-HR (95% CI)	*P*-value	RERI (95%CI)	AP (95%CI)	SI (95%CI)
**Overall (n = 17260)**						0.08 (0.02–0.14)	0.07 (0.05–0.10)	3.66 (1.37–7.25)
Normal	15375	12591	56960.76					
Left impaired alone	776	661	2864.18	1.01 (0.89–1.09)	0.645			
Right impaired alone	254	216	924.09	1.02 (0.88–1.18)	0.749			
Left + right impaired	855	758	2602.64	1.14 (1.04–1.24)	0.004			

Adjusted for age, sex, ethnicity, residence, living arrangement, education, marital status, smoking, alcohol consumption, regular exercise, BMI, cognitive function, hypertension, diabetes, heart disease, cerebrovascular disease, and respiratory disease.

ULF, Upper limb flexibility; HR, hazard ratios; CI, confidence interval; RERI, relative excess risk due to interaction; AP, attributable proportion due to interaction; SI, synergy index.
